# Supplementary material for: Innovative In Vivo Imaging and Single Cell Expression from Tumor Bulk and Corpus Callosum Reveal Glioma Stem Cells with Unique Regulatory Programs
Source: Cancers (Basel). 2025 Nov 30;17(23):3851. doi: 10.3390/cancers17233851 (PMC12691114; doi:10.3390/cancers17233851)
Supplement: Supplementary file 1 [file cancers-17-03851-s001.zip › cancers-3926208-Figure S1.pdf]

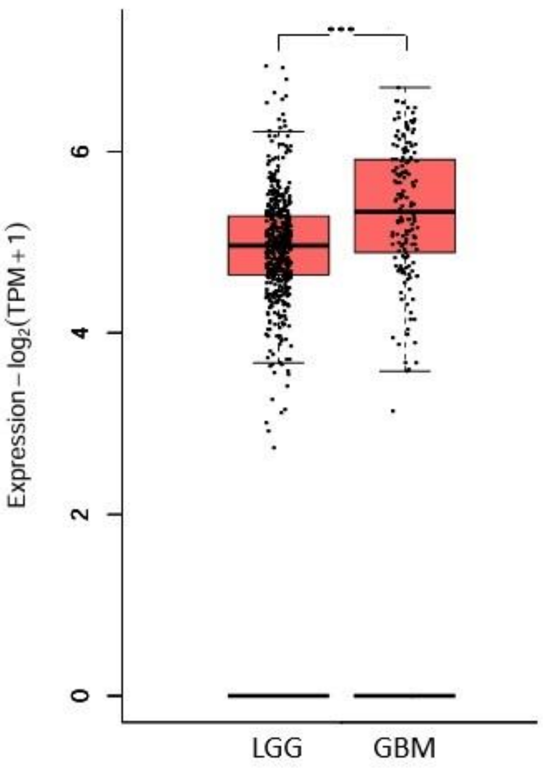

Supplementary Figure S1. Comparative expression of the CC-Iv gene signature between low-grade glioma (LGG) and glioblastoma (GBM) samples obtained from the TCGA dataset using the GEPIA2 platform (http://gepia2.cancer-pku.cn/, accessed on 20 October 2025). Box plot showing that CC-Iv signature expression is significantly higher in GBM than in LGG (1.7-fold increase, $p < 0.001$, t-test), supporting the constitutive activation of this signature in HGG.
